# Supplementary material for: Reclaiming motherhood through shame, distance, and gratitude—A phenomenological study of Swedish women’s lived experiences of giving birth while ill with COVID-19
Source: PLoS One. 2025 Oct 9;20(10):e0333937. doi: 10.1371/journal.pone.0333937 (PMC12510478; doi:10.1371/journal.pone.0333937)
Supplement: Attachment — Submitted filename: Response to Reviewers.docx [file pone.0333937.s003.docx]

# **Reviewer #1:** Review Comments on the Manuscript

Overall Evaluation
This manuscript presents a compelling and insightful qualitative study on the lived experiences of Swedish women who gave birth while ill with COVID-19. The phenomenological approach effectively captures the depth of participants' emotions and challenges, providing valuable insights for healthcare providers and policymakers. However, there are areas where the study could be strengthened, particularly in methodology, sample justification, thematic analysis, and integration with existing literature.

## ***Response:*** *Thank you for your positive feedback regarding our manuscript. We are grateful for your thorough review that is very helpful in improving the reporting of the study.*

# Strengths of the Manuscript The study addresses an underexplored area of maternal health during the COVID-19 pandemic. The use of a phenomenological reflective lifeworld approach allows for an in-depth exploration of lived experiences, contributing to the existing knowledge on maternal healthcare in crises. The study has clear public health implications, emphasizing the importance of emotional and psychosocial support for women giving birth under extraordinary conditions. Well-Structured Presentation The abstract is well-structured and provides a clear summary of the research, including objectives, methodology, key findings, and implications. The findings are logically presented under well-defined themes ("shame," "distance," and "gratefulness"), making it easy for readers to follow the emotional progression of participants' experiences. The discussion integrates findings with literature, making important connections between the experiences of these women and existing studies on childbirth, trauma, and the impact of COVID-19. Ethical Considerations The study follows rigorous ethical guidelines, securing approval from the Swedish Ethical Review Authority and ensuring informed consent from participants. The researchers acknowledge potential bias and discuss efforts to "bridle" preconceptions, which enhances the credibility of the qualitative findings. ***Response:*** *Thank you for acknowledging the study’s strengths.*

# Weaknesses and Areas for Improvement Sample Size and Diversity The study includes only 10 participants, which is relatively small even for qualitative research. While phenomenological studies prioritize depth over breadth, the study would benefit from: o Justifying why 10 participants were sufficient in capturing data saturation. ***R1: Response:*** *Thank you for your insightful question. You are correct in that phenomenological studies prioritize depth over breadth. Phenomenology according to Dahlberg et al is strongly linked to philosophy and does not use saturation, rather variation and depth of the phenomena. A usual sample size for this analysis is 8-12 participants, with 10 being typical. We have now clarified this in the methods section: The sample size was determined in line with phenomenological methodology, which prioritises depth of experience over breadth^1^. Please see line number 199-200.*

Discussing demographic variability (e.g., socio-economic background, education level, healthcare access) to ensure representativeness.
***R2: Response:*** *Thank you we have added the following text to the methods section: Participants represented a variety of obstetric and health-related backgrounds, including both primiparas and multiparas, and women with and without pregnancy complications. Please see line numbers 200-202.*

Addressing potential selection bias due to recruitment via hospital Instagram accounts, which may exclude women with limited internet access or digital literacy.
***R3: Response:*** *Thank you for this important comment. We have clarified that we recruited participants in two ways in the methods section, please see line 192-193. We have also added a statement regarding this limitation and its potential impact regarding transferability in the discussion: We also acknowledge that the part of the recruitment that took place via the hospital's Instagram account may have inadvertently excluded women with lower levels of digital literacy, which could in turn affect the transferability of the findings. (Please see, line numbers 592-595)*

## Methodological Rigor The study claims to use a phenomenological reflective lifeworld approach but does not provide enough detail on how researchers ensured rigor in analysis. o Were member checks or inter-coder reliability measures used to validate interpretations? Were any contradictory narratives encountered? If so, how were they reconciled in the thematic analysis? ***R4: Response:*** *Thank you. In phenomenology, the researcher interprets rather than describes the data (see* Dahlberg H, Dahlberg K. The question of meaning-a momentous issue for qualitative research. Int J Qual Stud Health Well-being. 2019 Dec;14(1):1598723), in pursuit of meaning. *Therefore, inter-coder reliability is not measured, nor is member-checking utilised. However, the analysis was continuously discussed with the senior author and checked against the text to make sure that the result was grounded in the data. Further, all authors confirmed the analysis and the essence in discussion. We have tried to describe this process as openly as possible in accordance with the SRQR checklist (see line numbers 618-619 as well as 605-609). Please specify if you have suggestions for details missing and we will gladly provide them.*

The use of NVivo for coding is mentioned, but it would be useful to describe how codes were developed and how emergent themes were validated.
***R5: Response:*** *Thank you for the question. NVivo was only used to organize the transcripts and find meaning units to form clusters. All analysis was done by the main author in discussion with the last author and no AI was used.*

Thematic Development
While the three core themes ("shame," "distance," and "gratefulness") are compelling, some nuances may be lost in broad categorization.
For example, "gratefulness" overlaps with the concept of "guilt," as women expressed gratitude despite their fears of burdening healthcare workers.
The theme of "distance" could be further broken down into physical, emotional, and communicative distance, each of which had unique implications for the birthing experience.
***R6: Response:*** *Thank you, we agree that there is an overlap between some of the constituents, this is common in phenomenology and is further described in the essence namely ‘Reclaiming motherhood through shame, distance and gratefulness’. We thank you for pointing out the difference in distance and have added this in the explanation of the essence: Therefore, the distance emerged as physical, emotional and communicative, which all impacted the birth experience.* (see line number 279-281)

Integration with Literature
While the discussion includes previous studies on childbirth and COVID-19, there is limited engagement with broader theories of maternal stress, trauma, and resilience.
Consider applying a conceptual framework (e.g., Lazarus & Folkman’s Cognitive Appraisal Model or Trauma-Informed Care) to contextualize the psychological responses observed in participants.
Comparison with studies from other countries would strengthen the discussion on cultural differences in pandemic maternity care.
***R7: Response:*** *Thank you for this excellent suggestion that truly adds to our findings. We have added a discussion about trauma-informed care, please see* *line numbers 572-580. Regarding the comparison with other countries: Unfortunately, there are very few articles writing about the topic, even from an international perspective. We have discussed the ones that we have found.*

Reflexivity and Researcher Influence
The role of the researcher in data collection and interpretation needs to be critically addressed.
The interviewer was not involved in clinical care, but what steps were taken to minimize power imbalances?
Did participants feel they could fully express negative experiences about healthcare settings, given that the researchers were affiliated with medical institutions?
***R8: Response:*** *Thank you for raising this important question. Several steps were taken to address the power imbalance in the interview setting. All interviews were conducted digitally, meaning that the participant was comfortable in her own home setting. The interviewer presented herself as a PhD-student, not as an obstetrician. She also used her own casual clothing, mostly hoodies or fleece jackets, and the background was neutral with no markings of a hospital setting. Please see line numbers 212-216.*

Suggested Revisions
Enhancing Methodological Transparency
Provide a stronger justification for the sample size, explaining how saturation was reached.

**Response:** please see R1 above.

Clarify coding procedures and validation strategies (e.g., multiple coders, intercoder reliability).
Discuss whether member-checking was used (e.g., did participants review summaries of their interviews to verify accuracy?).

***Response****: please see R4 above.*

Expanding Thematic Analysis
Consider refining thematic categories to avoid conceptual overlap:
Instead of "gratefulness," explore the tension between gratitude and guilt.
Instead of "distance," separate physical distance from emotional detachment.
Add counter-narratives or exceptions (e.g., did any women report positive aspects of giving birth while ill?).

***Response:*** *Please see R6 above.*

## Strengthening the Discussion Incorporate psychological and feminist theories on childbirth, trauma, and resilience. Compare findings with similar studies from other healthcare settings to provide a global perspective on maternity care during pandemics. ***Response:*** *Please see R7 above.*

## Reflect on how this study contributes to policymaking, particularly in crisis preparedness for maternal healthcare.

## ***R9: Response:*** *Thank you for addressing the need to further discuss contributions to policymaking, we have added this to the study’s conclusion. This study provides valuable insights for policymaking in strengthening crisis preparedness within maternal healthcare. Our findings highlight the need for policies that not only ensure physical safety and infection control but also protect women’s dignity, and emotional well-being during childbirth. In particular, the study underscores the importance of maintaining psychosocial support and respectful maternity care, even in the context of public health emergencies. Please see line numbers 633-636.*

## Addressing Reflexivity and Ethical Considerations Discuss the impact of researcher positionality on interview responses. Explain any steps taken to mitigate power imbalances between the interviewer and participants. Consider including a limitations section explicitly addressing biases and challenges in data collection. Addressing Reflexivity and Ethical Considerations. Explain any steps taken to mitigate power imbalances between the interviewer and participants. ***Response****: Please see R8 above.*

# Final Recommendation Major Revisions Required This study makes a valuable contribution to understanding the psychological and emotional impact of giving birth while ill with COVID-19. However, key methodological gaps need to be addressed to strengthen the credibility of findings. By refining thematic analysis, expanding discussion with theoretical frameworks, and improving methodological transparency, the paper can make a stronger impact on maternal healthcare policies and crisis preparedness strategies.

# **Reviewer #2:** Thank you for submitting this insightful manuscript on the lived experiences of Swedish women giving birth while ill with COVID-19. The study contributes valuable qualitative insights into the intersection of childbirth, illness, and healthcare provision during a pandemic. The phenomenological approach is well-justified, and the findings provide a nuanced understanding of the participants' experiences. Below are some suggestions to enhance clarity and conciseness in the manuscript.

## ***Response:*** *Thank you for your positive feedback regarding our manuscript. We are grateful for your thorough review that is very helpful in improving the reporting of the study.*

1. Redundancy in the Presentation of Participant Characteristics
The table detailing participant characteristics presents information that is already well described in the text. As the textual explanation provides a clearer understanding of the data, I recommend keeping the narrative and reducing the table to only non-redundant information. Alternatively, the table could be reformatted to emphasize key trends rather than listing all variables already covered in the text.

## ***Response:*** *Thank you, we agree that there was repetition. We have omitted the table all together since it did not convey any further information to the reader. Please see line number 246-259.* 2. Redundancy at the Beginning of the Results Section The introduction to the results section repeats some information already covered in the methodology and introduction. I suggest streamlining this part to focus directly on the key findings. A more direct transition to the "essence" of the phenomenon could improve readability and avoid repetition.

## ***Response:*** *Thank you. We have omitted the definitions of mild, moderate and severe covid to avoid repetition. The description of the essence of the phenomena needs to be deep and a few concepts of repletion of factors from the background are to be expected. Yet, we have tried to enhance clarity in the text.* 3. General Clarity and Conciseness While the phenomenological approach requires a rich narrative, there are sections where certain descriptions could be made more concise without losing depth. For example: - Some sections reiterate concepts of shame, guilt, and gratitude multiple times. Consolidating these discussions where appropriate could strengthen the impact of the findings.

## ***Response:*** *Thank you. We have tried to make the text more concise without losing meaning. If you have further concrete suggestions, it would be much appreciated.*

- The discussion could more explicitly connect the findings to practical recommendations for healthcare professionals, particularly in how to mitigate the emotional and psychological burden experienced by birthing women in crisis contexts.

## ***Response:*** *Thank you for this important point! We have added a discussion about trauma informed care and made sure to include care interventions to make it more useful for healthcare professionals. Please see response R7 to reviewer 1 above as well as line number 572-580.* 4. Data Availability Statement The current data availability statement notes that the data contain sensitive information and cannot be shared openly. While this is understandable, PLOS ONE requires clear justifications for any data-sharing restrictions. It may be helpful to specify if de-identified excerpts or aggregated data could be made available under controlled conditions.

## ***Response:*** *Thank you. We agree with the commitment to open science. Regrettably the Swedish ethical review authority has yet to provide guidelines regarding how qualitative data is to be shared openly. This strongly limits our possibility to share data openly.*

## ***Data Availability Statement:*** *Data contains potentially identifying and sensitive information, and there are ethical restrictions to sharing the data file as it includes individual interviews with participants. For data requests, please contact The Swedish Ethical Review Authority:* [registrator@etikprovning.se](mailto:registrator@etikprovning.se)*. Any specific requests concerning de-identified excerpts or aggregated data can be made directly to the corresponding author pending approval by the University of Gothenburg. Line numbers 640-646.* These revisions would enhance the clarity and impact of the manuscript while maintaining the depth of analysis. I appreciate the effort put into this study and believe that with these minor refinements, the manuscript will make a strong contribution to the field.

1.	Dahlberg K, Dahlberg H, Nyström M. Reflective Lifeworld Research. second edition ed. Lund: Studentlitteratur AB; 2008.
