# Supplementary material for: Reclaiming motherhood through shame, distance, and gratitude—A phenomenological study of Swedish women’s lived experiences of giving birth while ill with COVID-19
Source: PLoS One. 2025 Oct 9;20(10):e0333937. doi: 10.1371/journal.pone.0333937 (PMC12510478; doi:10.1371/journal.pone.0333937)
Supplement: Attachment — Submitted filename: Response to reviewers .docx [file pone.0333937.s004.docx]

**Reviewer #3**: Review Comments on the Manuscript

Reclaiming Motherhood through Shame, Distance, and Gratefulness - A Phenomenological Study of Swedish Women’s Lived experiences of giving birth while Ill with COVID-19

The primary aim of this study is to apply a phenomenological approach to explore the experiences of women who gave birth while infected with SARS-CoV-2 in Sweden. The study offers valuable insights from both health systems and policy perspectives. As social and healthcare practitioners are often required to navigate complex challenges in their work environments, the findings contribute meaningfully to our understanding of real-world practice. The study has undergone reviews. It does meet the requisite ethical and research standards. Some questions that arose are if the authors reflected on ‘bracketing or epoché’ though difficult to implement in practical sense. As traditional reliability and validity tools are not relevant in a phenomenological study. What were the team processes which enabled reflexivity into data-analysis? If the authors add a few more details into the data analysis, it would be useful to scholars of phenomenology approach.

Which language were the interviews conducted in? How did translation impact the data analysis? Thank you

*Response:* Thank you very much for your kind and encouraging feedback on our manuscript. We truly appreciate the time and care you devoted to reviewing our work. Your thoughtful comments have been very helpful in strengthening the clarity and reporting of the study, and we are sincerely grateful for your support.

In response to your questions, we have carefully revised the manuscript and addressed each point raised. Where appropriate, we have incorporated new clarifications into the manuscript and provided line references below.

*What were the team processes which enabled reflexivity into data-analysis? If the authors add a few more details into the data analysis, it would be useful to scholars of phenomenology approach.*

Thank you for this suggestion. We have added further details. Please see line number 147-151, 235-239, 641-647.

*Which language were the interviews conducted in? How did translation impact the data analysis?*

Thank you, this is an important point. All interviews were conducted in Swedish. Please see line number 207. We have now also included an explanation of our approach to translating the interview quotations. Please see line number 652-655.

**Reviewer #4:** This is a well-conceived and timely phenomenological study exploring women’s lived experiences of giving birth while ill with COVID-19 in Sweden. The revisions since the first review have strengthened the manuscript considerably, the methodology is now clearer, thematic analysis more nuanced, reflexivity better articulated, and the conclusion more firmly linked to crisis preparedness. The paper makes an important contribution to maternal health research in crisis contexts. With a few further refinements, the manuscript can achieve greater methodological transparency, conceptual precision, and integration with the broader qualitative literature.

*Response:* We sincerely thank you for your constructive, thoughtful, and generous feedback. We greatly appreciate the time and care invested in reviewing our manuscript. The comments have helped us to significantly enhance the methodological clarity, conceptual rigour, and practical relevance of the study.

In response to the suggestions, we have carefully revised the manuscript and addressed each point raised. Where appropriate, we have incorporated new clarifications into the manuscript and provided line references below.

Please find our point-by-point responses to each comment below.

Major Points

1. Trustworthiness and Rigor

While the reflective lifeworld approach and bridling are well explained, it would strengthen the manuscript to include a short paragraph mapping the study procedures explicitly to trustworthiness criteria (credibility, dependability, confirmability, transferability). For example: iterative immersion, analyst triangulation, and grounding in quotations (credibility); audit trail and reflexive discussions (dependability/confirmability); thick description of context and participant variation (transferability). Referencing Lincoln & Guba or Creswell’s validation strategies would enhance methodological transparency for readers less familiar with phenomenology.

*Response:* We are grateful for the reviewer’s thoughtful suggestion regarding Creswell’s validation strategies. While we recognize their importance in many qualitative traditions, we believe that directly applying these strategies would not be fully appropriate in the present study, as they are not grounded in phenomenological philosophy and may, in some respects, be at odds with its methodological commitments. Instead, we have followed the quality criteria articulated by Dahlberg, Dahlberg, and Nyström (2008), where rigour is ensured through openness, bridling of pre-understandings, and a systematic search for the essence and variations of lived experience. Phenomenology seeks to understand and describe the subjective experience of a phenomenon. To strengthen trustworthiness, we sought to describe the women’s lived experiences with openness, nuance, and methodological transparency. With respect to credibility, we have presented rich empirical excerpts to illustrate our interpretations. For confirmability, the identified essence and constituents were critically discussed and validated with co-authors who were not involved in the primary analysis. Regarding dependability, we ensured transparency by carefully documenting the analytic process, moving iteratively between whole and parts, and by engaging in reflexive discussions about our pre-understandings throughout the study.

We have incorporated parts of the above text into the discussion of the methodology section. We have also addressed the remaining points and integrated relevant reflections where they aligned with the structure and content of the manuscript.

Please see line number 606-614.

2. Thematic Precision

The subdivision of distance into physical, emotional, and communicative is excellent. However, gratefulness remains conceptually entangled with guilt and shame. Consider renaming this theme ambivalent gratitude or explicitly discussing the intertwining of gratitude and guilt in the Results section, to avoid interpretive ambiguity. The consistent use of gratitude rather than gratefulness is recommended.

*Response:* We sincerely thank the reviewer for this insightful observation. In response, we have revised the manuscript to reflect the suggested phrasing and now use gratitude consistently throughout the manuscript instead of gratefulness, to enhance conceptual clarity.

3. Integration with Broader Theory

The addition of trauma-informed care and stigma literature is valuable. To situate the findings more firmly, a short paragraph in the Discussion linking to Lazarus & Folkman’s stress–coping framework (appraisal, coping strategies) would be helpful, as would a brief reference to feminist childbirth literature (medicalization, agency, relational power). These connections would show that the insights extend beyond the pandemic context.

*Response:* We sincerely thank the reviewer for this thoughtful suggestion. We agree that Lazarus and Folkman’s stress–coping framework offers an important perspective for understanding how individuals appraise and respond to challenging situations. However, since our study is firmly situated within a phenomenological lifeworld approach, we have chosen to keep the discussion within this methodological and theoretical tradition to avoid diluting the phenomenological focus. We therefore respectfully refrain from adding this additional framework, but we greatly appreciate the insight and acknowledge its relevance for readers who may wish to interpret our findings through complementary theoretical lenses.

4. Reflexivity and Positionality

The reflexivity section is notably improved (non-clinical stance, casual clothing, digital interviews). To further strengthen it, one line acknowledging the unavoidable positionality associated with institutional affiliation in obstetric/midwifery contexts would be useful, along with a brief note on how this was mitigated (e.g., emphasizing confidentiality, explicitly inviting critique).

*Response:* Thank you for this thoughtful and constructive suggestion. We fully agree that acknowledging the unavoidable positionality linked to institutional affiliation is important in enhancing reflexivity and transparency. We have now added a sentence in the reflexivity section acknowledging the first author’s (MR) institutional affiliation in obstetrics and clarifying steps taken to mitigate this, including emphasising confidentiality, voluntary participation, and a bridled, open stance in interviews.

Please see line numbers 641 – 647

5. Policy and Practice Implications

The conclusion usefully links the findings to maternal healthcare preparedness in crises. To maximize the practical relevance, consider integrating implications more directly into the Discussion by including short practice pointers aligned with each theme (e.g., humanizing PPE, providing emotional reassurance, establishing clear protocols for early skin-to-skin contact, ensuring psychological follow-up for severe maternal illness).

*Response:* Thank you for this valuable and constructive suggestion. We have now integrated specific practice-oriented reflections into the Discussion section, in alignment with each theme. These additions include insights such as the importance of humanising care even when PPE is required, the therapeutic value of respectful presence, the significance of facilitating early skin-to-skin contact when feasible, and the need for structured psychological follow-up for severely ill mothers. We believe these refinements strengthen the study’s relevance for crisis preparedness in maternal healthcare

Please see line number: 541-543, 559-562, 572-574, 594-596.

Minor Points

- Abstract: revise phrasing to reclaiming motherhood while navigating shame, distance, and gratitude.

- Quotations: some excerpts are overly long (over eight lines). Please trim the longest to maintain analytic focus while preserving participants’ voices.

- Language/consistency: correct “it’s constituents” → “its constituents”; standardize % formatting (e.g., 80%); tidy hyphenation (post-birth, COVID-19 related).

- References: coverage is strong, but a final formatting pass for capitalization and URLs is recommended.

*Response:* Thank you for these helpful suggestions. In response, we have revised the abstract to reflect the suggested phrasing and now use gratitude consistently throughout the manuscript instead of gratefulness, to enhance conceptual clarity. We have reviewed the quotations and shortened those that exceeded eight lines, ensuring the analytic focus is preserved while retaining participants’ voices. Additionally, we have carefully edited the manuscript for improved language clarity and consistency, including correction of typographical errors (e.g., “it’s” to “its). A final review of the reference list was also conducted to ensure accuracy and formatting consistency.
